# Mesmerism Extraordinary

**Published:** 1893-02

**Authors:** 


### MESMERISM EXTRAORDINARY.

The following is from a Dalziel telegram, published in the Daily *Chronicle*, and other morning papers :

This morning, at the Charite Hospital, the series of experiments which are being made by Dr. Luys of the " exteriorization " of the human body were continued. Thanks to the kindness of Dr. Luys, a Dalziel representative was allowed to be present at the seance. So complete was the exteriorization of the subject that Dr. Luys was able to transfer a woman's sensibility into a tumbler of water. The tumbler was then taken out of sight of the hypnotized person, and the representative was invited to touch the water, and as his hands came in contact with it the woman started as if in pain. This experiment was repeated several times, the requisite precautions being taken that the hypnotized subject should not see the contact between the hands and the water. The water retained the sensibility a considerable time, and if drunk before the sensibility is exhausted, the patient falls into a deadly swoon. Dr. Luys was also able to confirm the wonderful discovery made by Colonel Roche, administrator of the Ecole Polytechnique, who found it was possible to transfer the sensibility of a hypnotized person to the negative of a photograph of the subject, and that the subject not only felt but showed signs of any mark made on the negative. Supposing, for instance, a scratch was drawn with a pin across the hand on the negative after it had been charged with sensibility, the subject would shriek with pain, and a few instants later a mark similar to that made on the negative would be visible on the hands of the subject. Dr. Luys tried the experiment to-day several times with an extraordinary sensitive subject now at the Charite, and each time with considerable success. The experiments are creating a great deal of interest in the scientific world.

